# SOX2 antagonizes WWC1 to drive YAP1 activation in esophageal squamous cell carcinoma

**DOI:** 10.1002/cam4.2569

**Published:** 2019-09-27

**Authors:** Yuhang Chai, Qihang Li, Hongying Zhao, Zhiyu Zhang, Xiaodan Yu, Lijuan Pang, Zheng Liu, Jin Zhao, Lianghai Wang, Feng Li

**Affiliations:** ^1^ Department of Pathology and Key Laboratory of Xinjiang Endemic and Ethnic Diseases/the First Affiliated Hospital Shihezi University School of Medicine Shihezi China; ^2^ Department of Pathology and Medical Research Center Beijing Chaoyang Hospital Capital Medical University Beijing China; ^3^ Department of Stomatology The First Affiliated Hospital Shihezi University School of Medicine Shihezi China

**Keywords:** ESCC, SOX2, WWC1, YAP1

## Abstract

Whether SOX2 and ACTL6A/TP63 interact with the Hippo‐YAP1 pathway in esophageal squamous cell carcinoma (ESCC) remains unclear. Here, we reveal that *SOX2*, *ACTL6A*, and *TP63* are co‐amplified and upregulated in ESCC samples. Multiple SOX2 binding peaks in the locus of WWC1, a Hippo‐YAP1 regulator, and an inverse correlation between the expression of SOX2 and WWC1 are identified, suggesting direct repression of WWC1 by SOX2. Expression scores of SOX2 are higher in tumors than normal tissues and positively correlated with nuclear YAP1 staining in primary ESCC. Moreover, SOX2 gain‐of‐function significantly promotes nuclear YAP1 expression in ESCC cells while silencing of SOX2 expression inhibits YAP1 activation. SOX2 overexpression leads to a significant enhancement of cell migration and invasion as well as chemoresistance to cisplatin, whereas knockdown of SOX2 or ectopic expression of WWC1 suppresses the SOX2‐induced migration ability and invasive potential. Disruption of this SOX2‐WWC1‐YAP1 axis could be a therapeutic strategy for SOX2‐dependent tumors.

## Graphical abstract

In the present study, we provide evidence that SOX2 is highly expressed in ESCC and activates YAP1 signaling by direct suppressing WWC1 transcription, thus promoting the migration, invasion, and drug resistance.

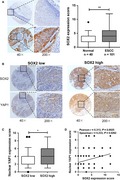

## 1 INTRODUCTION

Esophageal cancer is one of the most common gastrointestinal malignancies in the world. According to GLOBOCAN 2018, this disease ranks as the seventh most frequently diagnosed cancer (572 000 new cases) and the sixth leading cause of cancer death (509 000 deaths), with an estimated 1 in every 20 cancer deaths due to esophageal cancer.^1^ The two major histological types of esophageal cancer are esophageal squamous cell carcinoma (ESCC) and esophageal adenocarcinoma. ESCC accounts for 90% of patients with esophageal cancer worldwide, especially in the East, East Africa, and South America. The 5‐year survival rate of all patients with esophageal cancer is less than 20% even in developed countries.^2^ The occurrence of esophageal cancer is a multi‐factor, including environmental factors (such as smoking and drinking)^3^, ^4^ and genetic variants (such as chromosomal changes and methylation),^5^, ^6^ and multi‐step process.^2^ However, the detailed molecular mechanisms in ESCC development and progression remain to be fully elucidated.

SOX (SRY‐related HMG box) is a family of SRY (sex determination region of y chromosome) related genes, encoding a series of transcription factors involved in embryonic development and cellular regulation.^7^ SOX2 is a member of the SOX family B1 group and is located on chromosome 3q26.3~q27.^8^ Abnormal expression of SOX2 may be associated with human squamous cell carcinomas of the lung and esophagus (caused by amplification of the SOX2 locus),^9^, ^10^ human osteosarcoma,^11^ and melanoma.^12^ Moreover, the TP63 locus is frequently co‐amplified with SOX2, given that TP63 is located approximately 7 Mb from the SOX2 locus.^13^ SOX2 and p63 may be co‐localized on the genome in SCC and collaboratively regulate gene expression in squamous cell carcinoma.^13^ ACTL6A, which is located approximately 10 Mb apart on chromosome 3q, is frequently co‐amplified and co‐expressed with TP63 in a substantial proportion of head and neck squamous cell carcinomas (HNSCC).^14^

The Hippo signaling pathway is a potent regulator of cell proliferation, differentiation, and tissue homeostasis.^15^ The core components of the Hippo kinase/transcription modules are evolutionarily conserved. Yes‐associated protein 1 (YAP1) and its paralog transcriptional co‐activator with PDZ‐binding motif (TAZ; also known as WWTR1) are two major downstream effectors of the Hippo kinase cascade.^16^, ^17^ YAP1/TAZ function as transcriptional co‑activators that induce transcription of downstream cell‐proliferative and anti‐apoptotic genes via interactions with transcription factors, primarily TEA domain family members (TEAD) in the nucleus.^18^ Activation of the Hippo kinase cascade by various stimuli phosphorylate and inactivate YAP1 by triggering either the cytoplasmic retention or the degradation of YAP1, ultimately preventing the transcriptional output module.^15^ Kidney and brain expressed protein (KIBRA; also known as WWC1) acts as an upstream tumor suppressor that form a complex with neurofibromatosis 2 (NF2; also known as Merlin) to activate the Hippo kinase cassette and prevent YAP1 and TAZ activation.^19^, ^20^, ^21^

It has been reported that the Hippo pathway effector YAP1 is a direct transcriptional target of SOX2 in mesenchymal stem cells and osteoprogenitors^22^; WWC1 and NF2, two Hippo activators, appear to be directly repressed at the transcriptional level by SOX2 in human osteosarcomas.^23^ Moreover, ACTL6A and p63 could physically interact, cooperatively suppressing WWC1 transcription to activate the Hippo‐YAP1 pathway and thus promoting tumorigenesis in HNSCC.^14^ However, whether and how SOX2, ACTL6A, or TP63 interacts with the Hippo‐YAP1 pathway in ESCC remain to be determined.

Herein, we sought to identify the genetic alterations and expression profiles of related genes in ESCC. We have also used in vitro gain‐of‐function model to assess the regulation mechanism.

## 2 MATERIALS AND METHODS

### 2.1 Human tissue samples

A total of 101 ESCC and 40 non‐cancerous adjacent tissue samples were obtained from the First Affiliated Hospital of Shihezi University School of Medicine, Xinjiang Yili Prefecture Friendship Hospital, and the People's Hospital of Xinjiang Uyghur Autonomous Region in the years 2004‐2013. Informed consents were obtained from the patients, and the study was approved by the Ethics Committee of the First Affiliated Hospital, Shihezi University School of Medicine.

### 2.2 Immunohistochemistry

Immunohistochemical stainings of SOX2 and YAP1 were performed using primary antibodies against SOX2 (#2748, Cell Signaling Technology) and YAP1 (#14074, Cell Signaling Technology) on a BOND‐MAX Automated IHC/ISH Stainer (Leica) according to previously established protocols.^24^ Following staining, tissue microarray sections were dehydrated in graded alcohol, cleared in xylene, and mounted.

Immunostaining degree of each sample was scored as previously described by pathologists based on nuclear staining intensity (intensity score) and percentage of positive cells (extent score).^6^ The final immunoreactivity score for each case is the product of the intensity score and the extent score.

### 2.3 Cell culture

ESCC cell lines including Eca109, EC9706, TE‐1, and KYSE150 were purchased from the Cell Bank of Type Culture Collection of Chinese Academy of Sciences. All cells were maintained in RPMI 1640 medium supplemented with 10% fetal bovine serum at 37°C in a humidified incubator under 5% CO_2_ atmosphere. Eca109 cells stably expressing SOX2 (Eca109‐SOX2) were developed by transducing parental cells with SOX2 lentiviruses and selecting with 5 μg/mL Puromycin.

### 2.4 Western blotting

Total protein or nuclear and cytoplasmic fractions were isolated from cultured cells using RIPA buffer (#R0010) or a Nuclear and Cytoplasmic Protein Extraction Kit (#P0027, Beyotime Biotechnology) supplemented with protease inhibitors following the manufacturers’ instructions, respectively. Equal amounts of lysates were electrophoretically resolved and transferred to PVDF membranes. After blocking with 5% skimmed milk, the membranes were incubated with primary antibodies against SOX 2 (#2748, 1:500; Cell Signaling Technology), YAP1 (#14074, 1:1000; Cell Signaling Technology), TAZ (#4883, 1:1000; Cell Signaling Technology), *β*‐actin (1E9A3, 1:1000; ZSGB‐BIO), *α*‐tubulin (AF0001, Beyotime Biotechnology), Lamin B1 (AF1408, Beyotime Biotechnology), and appropriate peroxidase‐conjugated secondary antibodies. The signals were detected using enhanced chemiluminescence (Millipore).

### 2.5 Immunofluorescence

Cells cultured on glass coverslips were fixed with 4% paraformaldehyde followed by permeabilization with 0.1% Triton X‐100. Subsequently, cells were blocked with 1% BSA and incubated with YAP1 antibody, washed with PBS, incubated with Alexa Fluor 594‐Conjugated Goat anti‐Rabbit IgG (H + L) (#ZF‐0516, ZSGB‐BIO) as the secondary antibody, and counterstained with DAPI (#C1002, Beyotime Biotechnology). Images were taken at 60X magnification.

### 2.6 Transwell assay

Migration and invasion assays were carried out using Transwell chambers (Corning) according to our previous work.^25^ Migratory or invasive cells on the lower membrane surface were fixed, stained with 1% crystal violet and counted under a light microscope in five random fields.

### 2.7 Cell viability

Cell viability in the presence of increasing concentrations of cisplatin was measured using the Cell Counting Kit‐8 (CCK‐8) as previously described.^25^ Absorbance at 450 nm was measured with a microplate reader (BIO‐RAD xMark).

### 2.8 Xenograft assay

Five‐week‐old female BALB/C nude mice (Beijing Vital River Laboratory Animal Technology) were used in this study. One million KYSE150 cells grown at logarithm phase were subcutaneously injected into the armpit of mice to establish ESCC xenografts. Tumor volume was measured with a caliper and calculated using the formula length × width^2^ × *π*/6. The mice were sacrificed at the end of the experiments, and the tumors were excised and weighed. All procedures were performed with approval from the Animal Experimental Ethical Inspection of First Affiliated Hospital, Shihezi University School of Medicine.

### 2.9 Statistical analysis

Statistical analyses were performed using GraphPad Prism 7 or IBM SPSS Statistics. Pearson and Spearman's correlations were used to evaluate the significance of the association. Comparison between groups was conducted using a Mann‐Whitney U‐test or two‐tailed Student's *t* test. Numerical data were presented as means ± SEM unless stressed. A *P* value of <.05 was considered significant.

## 3 RESULT

### 3.1 *SOX2*, *ACTL6A*, and *TP63* are co‐amplified and upregulated in ESCC

**Figure 1:**
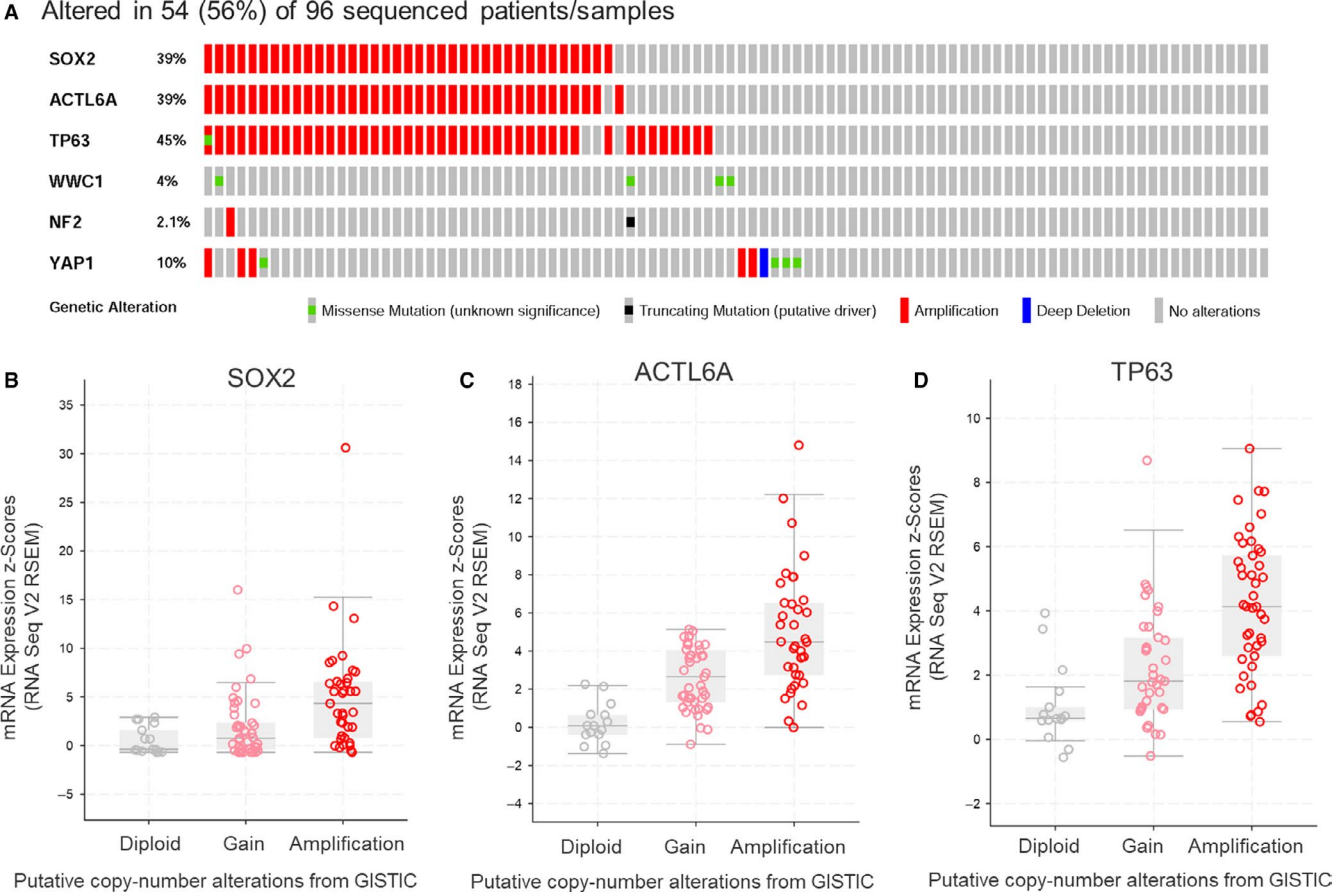
*SOX2* is amplified and upregulated together with *ACTL6A* and *TP63* in ESCC. (A) Gene copy‐number and mutation data from the TCGA for ESCC, showing frequent co‐amplification of SOX2, ACTL6A, and TP63. (B–D) *SOX2, ACTL6A, and TP63* mRNA expression correlate with their gene copy number, respectively. Data obtained from RNAseqV2 and GISTIC, respectively, of the TCGA ESCC (n = 96)

It has been previously suggested that SOX2 and ACTL6A/TP63 can control the activation of the Hippo‐YAP1 pathway. As a first step toward uncovering the functional contribution of *SOX2*, *ACTL6A*, and *TP63* to Hippo‐YAP1 pathway in ESCC, we analyzed the genetic alterations of ESCC patients from the Cancer Genome Atlas Network (TCGA). A substantial proportion of the samples (34 cases; 35.4%) exhibit co‐amplification of the *SOX2*, *ACTL6A*, and *TP63* loci, while *WWC1*, *NF2*, and *YAP1*, components of the Hippo‐YAP1 signaling, were barely mutated among these patients (Figure 1A). Moreover, expression of SOX2, ACTL6A, and TP63 correlated with their copy number status across ESCC tumors in the TCGA dataset, respectively (Figure 1B–D).

### 3.2 SOX2 mediates direct repression of the Hippo‐YAP1 regulator *WWC1* in ESCC

**Figure 2:**
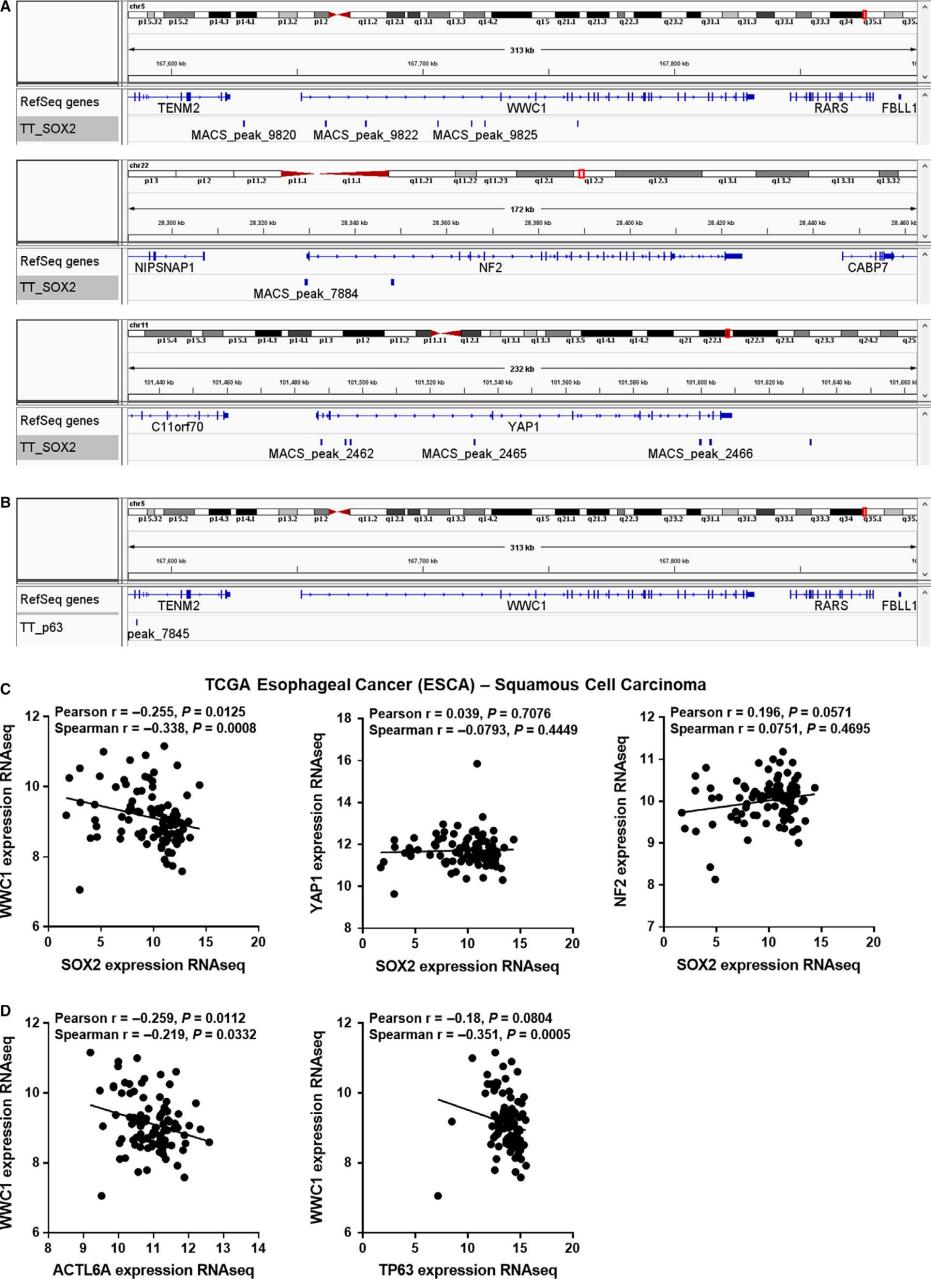
SOX2 is a direct transcriptional suppressor of the Hippo regulator *WWC1*. (A‐B) Trace from SOX2 (A) and p63 (B) ChIP‐seq data in ESCC cell line TT showing binding peaks around the indicated genes. (C) Correlation between SOX2 and WWC1, YAP1, and NF2 in SCC samples from the TCGA Esophageal Cancer (ESCA). (D) Correlation between ACTL6A, TP63, and WWC1 in SCC samples from ESCA dataset

To understand the transcriptional regulation involved in the YAP1 activation in ESCC, we first assessed chromatin immunoprecipitation/high‐throughput sequencing (ChIP‐seq) data of endogenous SOX2 and p63 in ESCC cell line TT, then integrated with RNA‐seq data from human ESCC in the TCGA dataset (Figure 2). Intriguingly, ChIP‐seq data for SOX2 binding showed multiple SOX2 binding peaks in the loci of *WWC1*, *NF2*, and *YAP1* (Figure 2A). However, among these candidate transcription targets, significant inverse correlation with *SOX2* expression was only identified for *WWC1* (Pearson *r* = −.255, *P* = .0125; Spearman *r* = −.338, *P* = .0008), but not for *NF2* and *YAP1* (*P* > .05; Figure 2C). Although a reverse trend between *ACTL6A*/*TP63* and *WWC1* was observed in the in vivo context (Figure 2D), there was no binding site of p63 to the *WWC1* locus (Figure 2B). Together, these findings suggest *WWC1* locus being a direct target of SOX2.

### 3.3 SOX2 is overexpressed and controls YAP1 activity in ESCC

**Figure 3:**
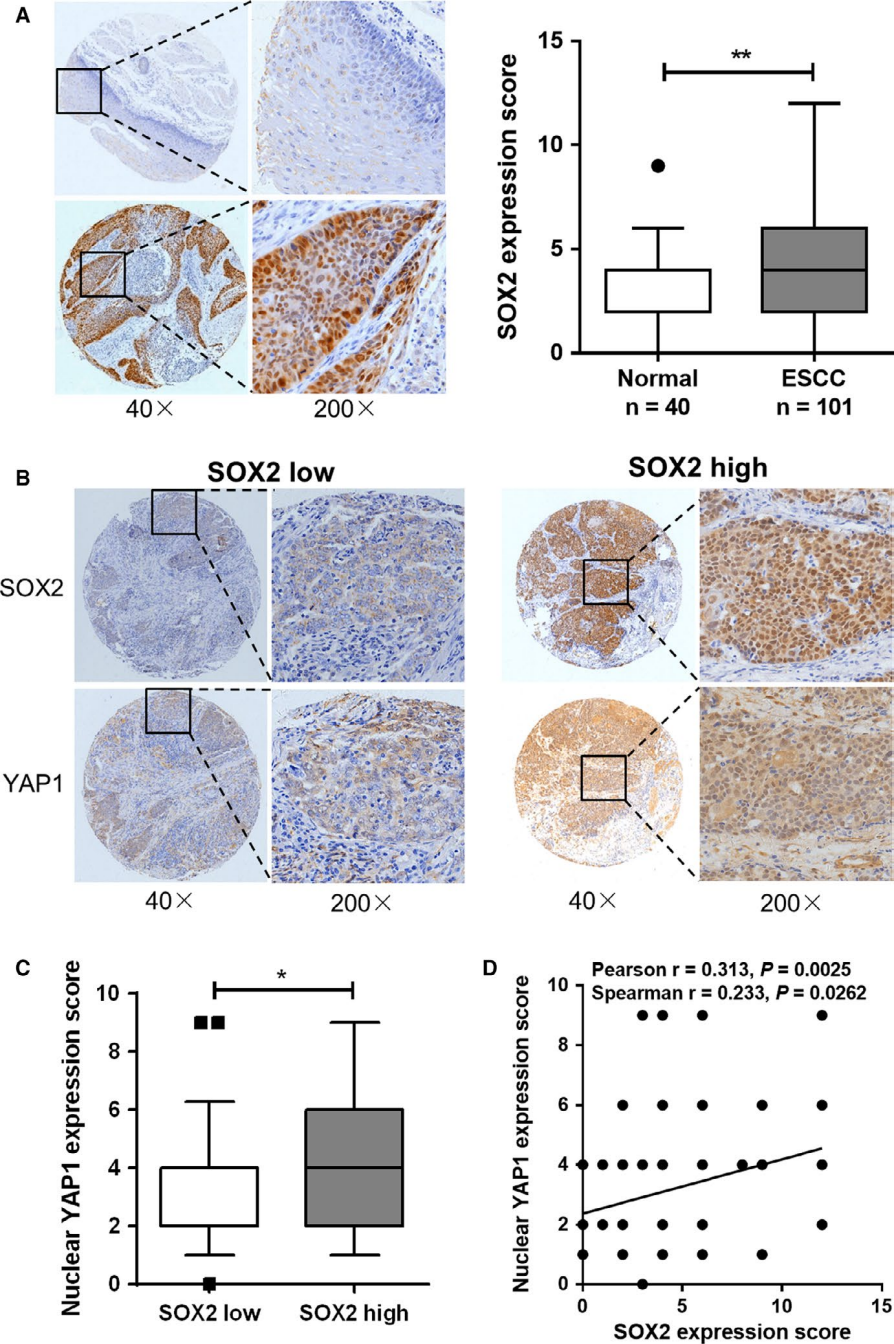
SOX2 regulates YAP1 activity in vivo in ESCC. (A) Representative immunohistochemistry staining and statistical analysis of SOX2 expression in a panel of 101 primary ESCC and 40 adjacent normal squamous epithelium tissues. (B) Representative images of YAP1 expression measured by immunohistochemistry in SOX2 high and SOX2 low ESCC samples. (C) Statistical analysis of nuclear YAP1 expression score in SOX2 high and SOX2 low ESCC samples. The cutoff value for high vs low SOX2 levels was set at the median. (D) Scatterplot representing the correlation between nuclear YAP1 and SOX2 expression scores in the ESCC cohort (n = 91). *P < .05, **P < .01

To provide further evidence for the control of Hippo‐YAP1 signaling pathway mediated by SOX2 in human ESCC, we characterized SOX2 and YAP1 expression by immunohistochemistry staining in a tissue microarray derived from an ESCC cohort of 101 patients. Results showed that SOX2 protein was mainly located in the nucleus of ESCC cells and the expression scores of SOX2 in ESCC were significantly higher than that in adjacent normal tissues (Figure 3A). Among them, 40 cases (40/101, 39.6%) of ESCC showed high expression score (>median) of SOX2 protein, whereas only 5 cases (5/40, 12.5%) of normal tissues exhibited high SOX2 expression. Stronger immunosignal in the nucleus of tumor cells was also observed for YAP1.^6^ Importantly, we found increased nuclear YAP1 staining in primary ESCC specimens with high levels of SOX2 (Figure 3B). Quantification of this series of samples showed that nuclear YAP1 expression was higher in ESCC with high SOX2 scores than that with low expression of SOX2 (Figure 3C). Furthermore, nuclear YAP1 expression score was positively associated with elevated SOX2 (Pearson *r* = .313, *P* = .0025; Spearman *r* = .233, *P* = .0262; Figure 3D).

**Figure 4:**
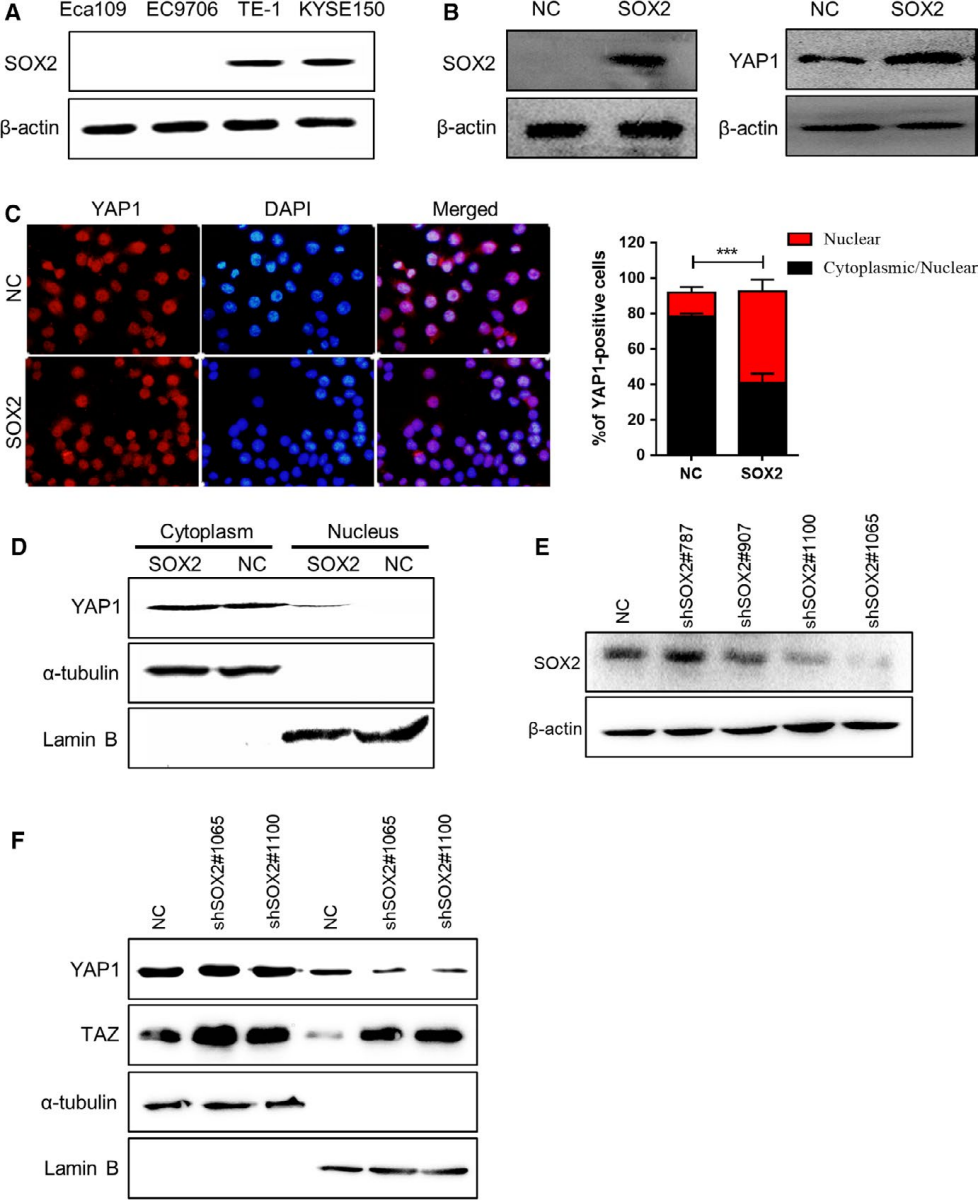
SOX2 controls endogenous YAP1 localization in ESCC cells. (A) Western blot analysis of endogenous SOX2 expression in a panel of ESCC cell lines. *β*‐Actin was served as a loading control. (B) Immunoblot analysis for SOX2 and total YAP1 protein levels after SOX2 overexpression in Eca109 cells. (C) Representative immunofluorescence staining and statistical analysis of YAP1 expression for the percentage of cells staining nuclear or both nuclear/cytoplasmic in Eca109‐vector and Eca109‐SOX2 cells. (D) Western blotting for nuclear YAP1 expression after SOX2 overexpression in Eca109 cells. *α*‐tubulin and Lamin B were used as loading controls. (E) Immunoblot analysis of SOX2 expression in KYSE150 cells after treatment with different shRNAs against SOX2. *β*‐actin was used as a loading control. (F) Western blotting for nuclear YAP1 and TAZ expression after SOX2 knockdown in KYSE150 cells. *α*‐tubulin and Lamin B were served as loading controls

Next, we sought to determine whether SOX2 gain‐of‐function was associated with YAP1 regulation in ESCC cells. Among a panel of ESCC cell lines, the expression of endogenous SOX2 protein was low in Eca109 and EC9706 cells (Figure 4A). Eca109 cells inoculated with SOX2‐overexpressing lentivirus (Eca109‐SOX2) showed higher expression of SOX2 protein than cells treated with a lentiviral vector (Eca109‐vector) as expected. The expression of total YAP1 protein increased after overexpression of SOX2 in Eca109 cells as detected by Western blotting (Figure 4B). Remarkably, immunofluorescence assay for YAP1 showed that the percentage of Eca109 cells staining nuclear YAP1 was significantly increased following overexpression of SOX2 (Figure 4C). We also analyzed YAP1 localization by immunoblot analysis of fractionated lysates from ESCC cells. SOX2 overexpression led to increased nuclear YAP1 level in Eca109‐SOX2 cells, while YAP1 was mainly in the cytoplasm in parental cells (Figure 4D). Conversely, knockdown of SOX2 expression by shRNAs in KYSE150 cells inhibited the nuclear levels of YAP1, but not TAZ (Figure 4E,F), confirming the control of YAP1 localization by SOX2.

### 3.4 SOX2 overexpression promotes aggressive phenotypes that can be antagonized by WWC1

**Figure 5:**
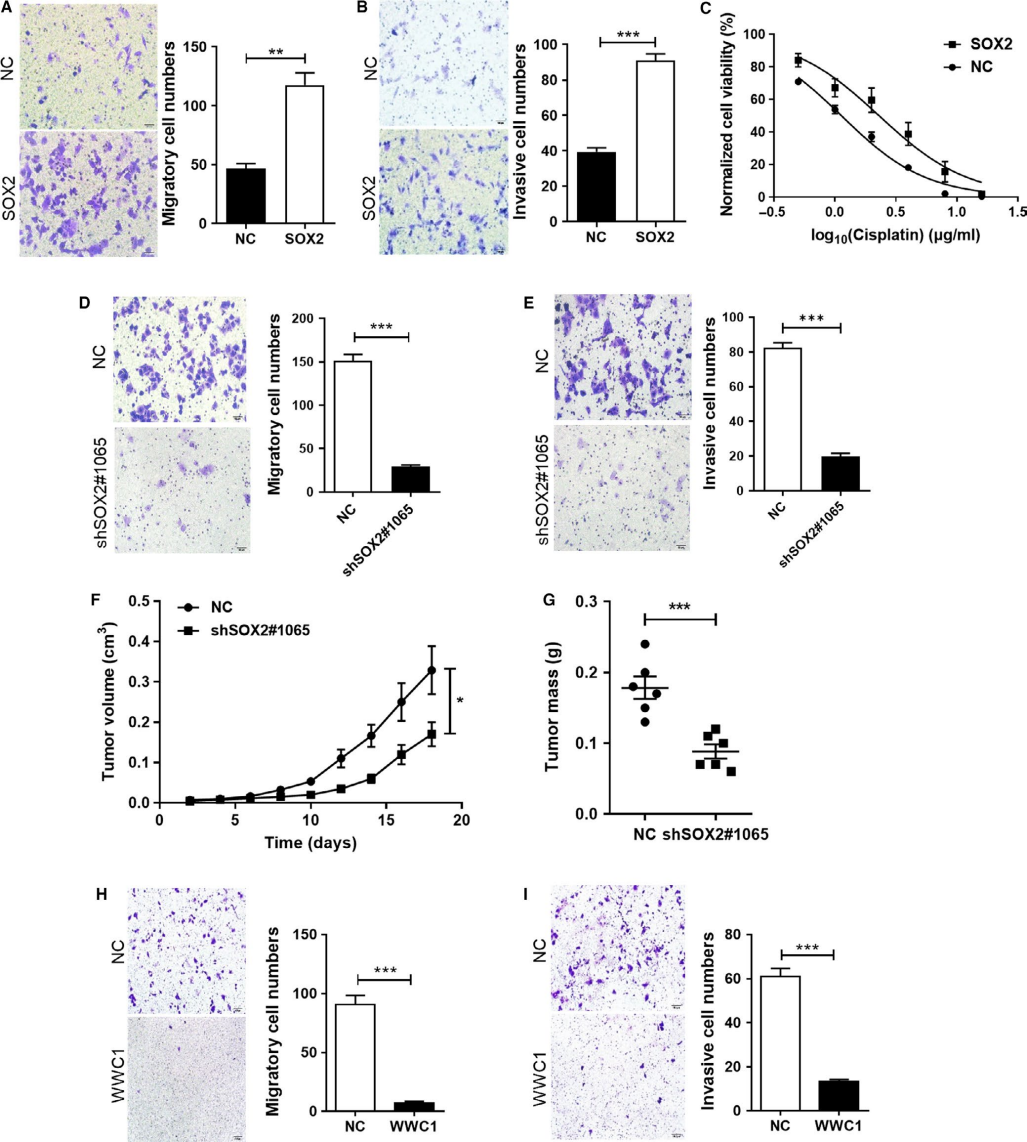
SOX2 overexpression drives migration and invasion that can be antagonized by WWC1. (A–B) Effect of SOX2 on cell migration (A) and invasion (B) of Eca109 cells assessed using Transwell assay. (C) Dose‐response curves of Eca109‐SOX2 and Eca109‐vector cells to cisplatin. (D–E) Effect of shSOX2 on cell migration (D) and invasion (E) of KYSE150 cells. (F‐G) KYSE150‐derived xenograft model was established in nude mice (n = 6 per group). Tumor volume was measured every other day (F), and tumor weights were measured at the end of the experiment (G). (H‐I) Effect of WWC1 on cell migration (H) and invasion (I) of Eca109‐SOX2 cells assessed using Transwell assay. *P < .05, **P < .01, ***P < .001

YAP1 is pervasively activated in human cancers including ESCC, where its activation is required to instruct malignant properties, such as chemoresistance and metastasis.^6^, ^26^ To determine the effect of SOX2 on the motility of ESCC cells, Transwell assays were performed. Results showed that overexpression of SOX2 in Eca109 cells resulted in a significant enhancement of their migration ability and invasive potential compared to control groups (Figure 5A,B). Furthermore, we found that enhanced SOX2 expression in Eca109 cells promoted chemoresistance to cisplatin, one of the most frequently used chemotherapeutic drug for esophageal cancer, as demonstrated by a shift in the IC_50_ (Figure 5C). On the contrary, silencing of SOX2 by shRNA markedly reduced migration and invasion of KYSE150 cells (Figure 5D,E). We next established a xenograft model in nude mice to address the role of SOX2 in tumor growth. Knockdown of SOX2 potently suppressed KYSE150‐derived xenograft growth, as measured by tumor volume and tumor weight (Figure 5F,G). To test the potential contribution of WWC1 in ESCC, we also performed gain‐of‐function experiments. Overexpression of the repressed WWC1 by a plasmid^27^ in Eca109‐SOX2 cells potently abrogated the SOX2‐induced migration and invasion (Figure 5H,I), providing evidence that repression of WWC1 is required for the functionality of SOX2 in ESCC.

## 4 DISCUSSION

Recent evidence suggests that SOX2 and ACTL6A/p63 may regulate Hippo pathway components in osteosarcoma and HNSCC, respectively.^14^, ^23^ In this report, we show that *SOX2*, *ACTL6A*, and *TP63* are co‐amplified and upregulated in ESCC samples. Through integrating genomic analysis and transcriptome profiling of these transcription factors, we outline a pathway in which SOX2‐mediated direct repression of the Hippo regulator WWC1 in ESCC. SOX2‐promoted YAP1 activation is confirmed both in clinical samples and ESCC cells. Of note, while SOX2 is supposed to bind the 3′ untranslated portion of WWC1 mRNA in osteosarcoma,^23^ multiple SOX2 binding peaks are identified in the upper region of *WWC1* in ESCC TT cells, suggesting SOX2 can regulate the Hippo‐YAP1 signaling in a context‐dependent manner. It is noteworthy that regulation of TAZ by SOX2 is distinct from that of YAP1 in ESCC cells, although YAP1 and TAZ are often described to be equivalent downstream of the Hippo pathway. Consistent with this finding, TAZ expression is not decreased in SOX2‐depleted osteoprogenitors.^22^ Furthermore, YAP1 shows different functional roles compared to TAZ in osteosarcoma and hepatocellular carcinoma.^22^, ^23^, ^28^

SOX2 has been implicated in tumorigenicity, drug resistance, and metastasis in at least 25 human cancers.^29^ Regarding clinical prognosis for cancer patients, high SOX2 expression has been linked to poor prognosis and increased metastatic capacity in the majority of cancers, such as ESCC^30^, ^31^ and breast cancer.^32^, ^33^ However, a few studies on the role of SOX2 in cancer development have led to contrasting findings: low or negative SOX2 expression is associated with worse prognosis in at least four cancers,^29^ including gastric cancer,^34^, ^35^ squamous cell lung cancer,^36^ and ESCC.^37^, ^38^ Given the conflicting reports regarding SOX2 expression and patient prognosis, there are clear needs for further investigation into the clinical implications of SOX2, particularly how SOX2 influences tumor progression. SOX2 has been described to promote tumor growth through activation of the AKT/mTORC1 signaling^39^ and to promote metastasis by activating the STAT3/HIF‐1*α* pathway.^40^ Targeted silencing of SOX2 by an artificial transcription factor shows an anti‐tumor effect in ESCC.^41^ In the present study, ectopic SOX2 expression promotes migration, invasion, and drug resistance of ESCC cells, while knockdown of SOX2 or WWC1 overexpression diminishes their migration ability and invasive potential.

In summary, SOX2 is highly expressed in ESCC and activates YAP1 signaling by direct suppressing *WWC1* transcription, thus promoting the migration, invasion, and drug resistance. This SOX2‐WWC1‐YAP1 axis in ESCC may serve as a target for cancer therapy.

## CONFLICTS OF INTEREST

The authors declare no conflict of interest.

## Data Availability

The data that support the findings of this study are available from the corresponding author upon reasonable request.
